# The Potential Impact of Age on Gut Microbiota in Patients with Major Depressive Disorder: A Secondary Analysis of the Prospective Observational Study

**DOI:** 10.3390/jpm12111827

**Published:** 2022-11-03

**Authors:** Katsuma Miyaho, Kenji Sanada, Shunya Kurokawa, Arisa Tanaka, Tomoyuki Tachibana, Chiharu Ishii, Yoshihiro Noda, Shinichiro Nakajima, Shinji Fukuda, Masaru Mimura, Taishiro Kishimoto, Akira Iwanami

**Affiliations:** 1 Department of Psychiatry, Showa University School of Medicine, 6-11-11 Kitakarasuyama, Setagaya-ku, Tokyo 157-8577, Japan; 2 Department of Neuropsychiatry, Keio University School of Medicine, 35 Shinanomachi, Shinjuku-ku, Tokyo 160-8582, Japan; 3 Institute for Advanced Biosciences, Keio University, 246-2 Mizukami, Kakuganji, Tsuruoka 997-0052, Yamagata, Japan; 4 Gut Environmental Design Group, Kanagawa Institute of Industrial Science and Technology, 3-25-13 Tonomachi, Kawasaki 210-0821, Kanagawa, Japan; 5 Transborder Medical Research Center, University of Tsukuba, 1-1-1 Tennodai, Tsukuba 305-8575, Ibaraki, Japan; 6 Laboratory for Regenerative Microbiology, Graduate School of Medicine, Juntendo University, 2-1-1 Hongo, Bunkyo-ku, Tokyo 113-8421, Japan

**Keywords:** gut microbiota, major depressive disorder, aging, gastrointestinal symptoms, prospective study

## Abstract

We aimed to investigate the impact of aging on the relationship among the composition of gut microbiota, gastrointestinal (GI) symptoms, and the course of treatment for major depressive disorder (MDD) by analyzing the datasets from our previous study. Patients with MDD were recruited, and their stools were collected at three time points (baseline, midterm, and endpoint) following the usual antidepressant treatment. Gut microbiota were analyzed using 16S rRNA gene sequencing. Patients were categorized into two groups based on their age: the late-life group over 60 years and the middle-aged group under 60 years. GI symptoms were assessed with scores of item 11 of the Hamilton Anxiety Rating Scale. One hundred and ninety samples were collected from 32 patients with MDD. Several gut microbes had higher relative abundances in the late-life group than in the middle-aged group. In addition, the late-life group showed significantly higher diversity in the Chao1 index at baseline compared with the middle-aged group. We further found possible microbial taxa related to GI symptoms in patients with late-life depression. The abundance of several bacterial taxa may contribute to GI symptoms in the late-life depression, and our findings suggest that the therapeutic targets for the application of gut microbiota may differ depending on the age group of patients with depression.

## 1. Introduction

About 38 trillion gut microbial cells exist in the human intestinal tract [1] and compose an extremely complicated bacterial system, which not only plays an important role in the immune system but also in the functioning of the brain–gut axis [2]. In addition, gut microbiota are influenced by physiological factors, such as diet and lifestyle, that can rapidly alter their composition [3]. Consequently, the features of human gut microbiota change during the aging process. Previous studies [4,5,6] report compositional changes in gut microbiota with age in healthy subjects within the three-year period after birth, while interpersonal compositional variations were smaller among adults in comparison with children.

Major depressive disorder (MDD) is a common mental disorder and the third leading cause of years lived with disability (YLD) rates [7]. Many preclinical studies have revealed a so-called “brain–gut interaction” in which changes in the gut microbiota affect the central nervous system, suggesting that this may contribute to the pathophysiology of MDD [2,8]. Patients with MDD have various symptoms including depressive mood and loss of interest. In general, it is clinically well known that patients with MDD, specifically older adults, often experience gastrointestinal (GI) symptoms with prevalence rates of up to 64.7% in the primary care setting [9]. Similarly, a meta-analysis noted that older patients with depression experienced more GI symptoms compared with younger adult patients with depression [10].

However, to the best of our knowledge, no studies investigated the impact of aging on the association between the composition of gut microbiota and GI symptoms in patients with MDD. Thus, we reanalyzed the data from our original study [11] for a different objective to conduct a preliminary study to examine the influence of age on the brain–gut interactions in patients with MDD. This study aimed to investigate the composition of gut microbiota in patients with MDD stratified based on age in a naturalistic treatment course; specifically, we divided the patients with MDD into the middle-aged and the late-life groups (<60 years and ≥60 years, respectively). We also examined the relationship between GI symptoms and related bacterial taxa in both groups. Our hypotheses were that there would be differences in bacterial features between the two groups and that several bacterial taxa would be related to GI symptoms in the late-life group.

## 2. Materials and Methods

### 2.1. Study Design

The present investigation was a secondary analysis of our multicenter prospective observational study that had examined whether various classes of psychotropics affect gut microbiota in patients with MDD and anxiety disorders [11]. The present analysis aimed at investigating the association between compositions of gut microbiota and age in inpatients and outpatients with MDD during antidepressant treatment as usual in clinical settings. This trial was registered with the UMIN Clinical Trials Registry (UMIN000021833) and was approved by the Ethical Committee of Showa University Karasuyama Hospital and Keio University School of Medicine. All patients were informed about the purposes and procedures of the study thorough careful explanation and provided written consent. Detailed information was reported elsewhere [11].

### 2.2. Participants

The study participants were recruited between June 2017 and January 2018 at Showa University Karasuyama Hospital, Keio University Hospital, and Komagino Hospital in Tokyo. The inclusion criteria were adult patients aged 20 years or older meeting the criteria for MDD in the *Diagnostic and Statistical Manual of Mental Disorders*, 5th edition (DSM-5), who were treated with psychotropics including antidepressants and/or antipsychotics. The exclusion criteria were: (1) those with any organic GI disorders; (2) those taking antibiotic medication at any time during the study; or (3) those whose psychiatric symptoms might worsen by participating in the study.

### 2.3. Study Procedure

For inpatients, fresh stool samples were collected, and psychiatric assessments were performed at three time points during the hospitalization. Baseline assessments (BL) were conducted within 10 days of admission; midterm assessments (T1) were completed between 14 and 20 days after admission, and endpoint assessments (T2) were carried out 21 days after admission and before discharge. A period of at least one week marked an evaluation interval. For outpatients, fresh stool samples and psychiatric assessments were performed at three consecutive outpatient visits corresponding to BL, T1, and T2.

### 2.4. Fecal Collection and Psychiatric Assessments

Fecal samples were collected per each time point and stored at −80 °C until the analysis. The mean value of data was used when two or three fecal samples were collected at each time point. Comprehensive psychiatric assessments comprised the Hamilton Depression Scale (17 items) (HAM-D) [12] and Hamilton Anxiety Scale (HAM-A) [13], which were administered by trained psychiatrists and psychologists.

### 2.5. Gastrointestinal (GI) Assessments

We used scores of item 11 of the HAM-A for GI symptoms. The HAM-A is a scale for measuring the severity of anxiety, consisting of 14 items, each rated on a scale of 0 (not present) to 4 (severe), with a total score ranging from 0 to 56 [14].

### 2.6. Classification of Patients

We divided patients into the two groups based on their age: ≥60 years (late-life group) or <60 years (middle-aged group). As mentioned above, since geriatric patients with MDD often present GI symptoms, we first divided patients into the two groups based on their scores of the HAM-A for GI symptoms: GI-present group or GI-absent group. Likewise, we further classified the late-life group (*n* = 14) into the two subgroups: GI-present group (*n* = 8) and GI-absent group (*n* = 6).

### 2.7. Sample Analysis

Fecal samples were immediately frozen after collection and transported to our departments (Showa University Karasuyama Hospital or Keio University Hospital) within 48 h. They were kept in a freezer at −80 °C for further analyses. The 16S rRNA gene was analyzed by the following method (for details, see [15]). Firstly, fecal samples were lyophilized for approximately 12–18 h using a VD-800R lyophilizer (TAITEC, Nagoya, Aichi, Japan). Each freeze-dried fecal sample was combined with four 3.0 mm zirconia beads and subjected to vigorous shaking (1500 rpm for 10 min) using a Shake Master (Biomedical Science, Shinjuku, Tokyo, Japan). Secondly, approximately 10 mg of each fecal sample was combined with approximately 100 mg of 0.1 mm zirconia/silica beads, 300 μL DNA extraction buffer (TE containing 1% (*w/v*) sodium dodecyl sulfate), and 300 μL of phenol/chloroform/isoamyl alcohol (25:24:1) and subjected to vigorous shaking (1500 rpm for 5 min) using a Shake Master. The resulting emulsion was subjected to centrifugation at 17,800× *g* for 10 min at room temperature. RNA was removed from the sample by RNase A treatment from bacterial genomic DNA purified from the aqueous phase. The resulting DNA sample was then purified again by another round of phenol/chloroform/isoamyl alcohol treatment and ethanol precipitation by GENE PREP STAR PI-480 (Kurabo Industries Ltd., Osaka, Japan). The V1-V2 hypervariable region of 16S rRNA-encoding genes were amplified by PCR using a bacterial universal primer set [16,17]. The amplicons were analyzed using a Miseq sequencer (Illumina, San Diego, CA, USA) with some modifications previously indicated [15]. Filter-passed reads were processed using the Quantitative Insights into Microbial Ecology (QIIME) 2 (2019.10) [18]. Denoising and trimming of sequences were processed using DADA2.20 bp, and 19 bp reads were trimmed from 5′ ends of forward and reverse reads, respectively, to remove primer sequence. Some 280 bp and 210 bp length reads from 5′ ends were used for further steps. Sequences were clustered into operational taxonomic units (OTUs) that reached 97% nucleotide similarity, and OTUs were assigned to the SILVA132 database [19,20] using the Naive Bayesian Classifier algorithm. Alpha diversity of gut microbiota was analyzed using Chao1 and Shannon indices. Principal coordinate analysis (PCoA) based on UniFrac distance and analysis of similarity (ANOSIM) tests was conducted using the QIIME 2. Differences in OTU abundance at the family and genus levels between groups were identified using Linear Discriminant Analysis (LDA) Effect Size (LEfSe) [21]. The longitudinal changes for each taxon were examined using the absolute value of the difference at two points: baseline (BL) and midterm (T1) and baseline (BL) and endpoint (T2). LEfSe combines the tests for statistical significance (Kruskal–Wallis test and pairwise Wilcoxon test) with LDA.

### 2.8. Statistical Assessments

We conducted data analyses using IBM SPSS Statistics version 25.0 (SPSS Inc. Chicago, IL, USA). Continuous and categorical variables were described as the mean ± standard deviation (SD) and number (%), respectively. All variables were inspected to test the data distribution using histograms, q-q plots, and Kolmogorov–Smirnov tests before conducting statistical analyses. Independent *t*-tests were used to compare differences in demographics and clinical characteristics at baseline and alpha diversity (Chao1 and Shannon indices) at baseline and endpoint between the late-life group and middle-aged group, as well as GI symptoms of the HAM-A between the late-life and middle-aged groups (Table 1). A categorical variable (i.e., sex) was compared between the groups using Pearson’s Chi-squared test. Based on our hypotheses, a significance level was set to 0.05 for all statistical tests. LEfSe analysis was performed under the following conditions: the alpha value for the factorial Kruskal–Wallis test among classes was 0.05, while the threshold on the logarithmic LDA score for discriminative features was 3.0. LEfSe analysis was used to identify microbial taxa, which were differentially abundant between the late-life and middle-aged groups and to analyze the changes in each microbial taxon (BL and T1 and BL and T2). A one-way ANCOVA was conducted to determine a statistically significant difference between the late-life and middle-aged groups on each bacterial taxon with significant differences in LEfSe controlling for GI symptom scores. In cases where there was a significant difference in each taxon in either the late-life group or the middle-aged group, Mann–Whitney U-test was performed to evaluate the difference in each taxon, and statistical corrections were conducted by controlling the false discovery rate (FDR) through the Benjamini–Hochberg procedure [22] with alpha set at 0.05. As exploratory analyses, Spearman’s correlation coefficients were calculated between microbial taxa with significant differences and clinical symptoms. The relationship between HAM-D scores and microbial taxa was also determined by calculating Spearman’s correlation coefficients. In cases where there was a significant correlation in each taxon, independent *t*-tests were performed to compare differences between the late-life and middle-aged groups.

## 3. Results

**Figure 1:**
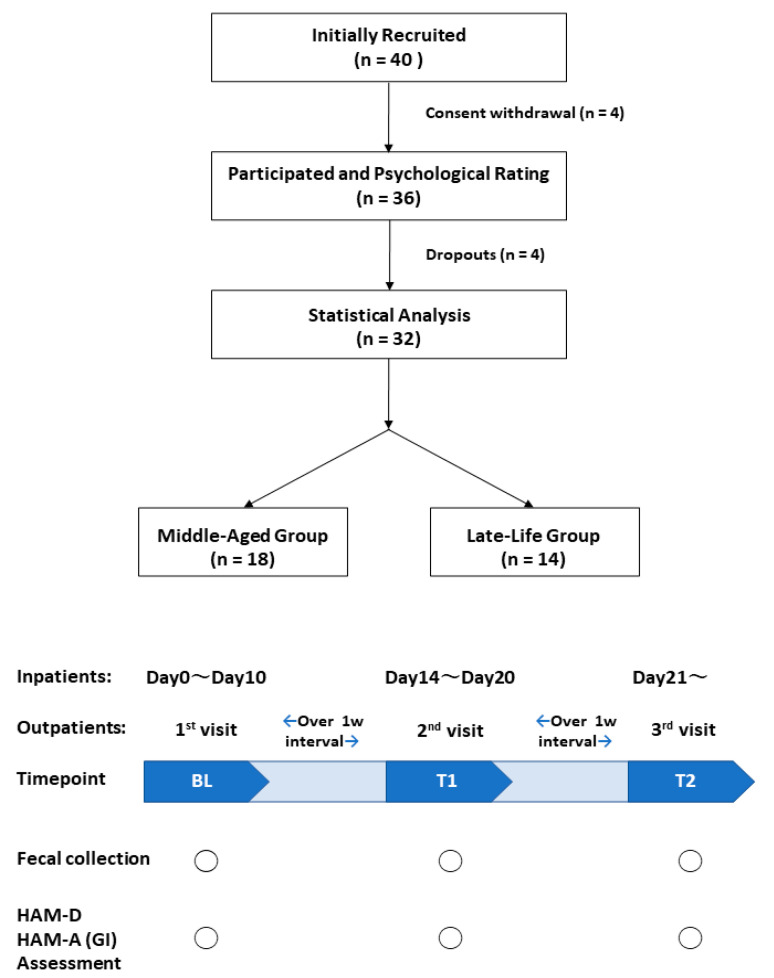
Flow chart of participants through the trial. BL, baseline assessments; T1, midterm assessments; T2, endpoint assessments; HAM-D, Hamilton Depression Scale; HAM-A, Hamilton Anxiety Scale; GI, gastrointestinal.

The study flow chart is shown in Figure 1. Of the 40 patients who were initially recruited as inpatients and outpatients, we included 32 patients (17 females (53.1%) and 12 inpatients (37.5%)) in this study who had completed at least one clinical assessment, resulting in 188 fecal samples analyzed (BL, 64 samples; T1, 64 samples; and T2, 60 samples). Of the included 32 patients, five patients did not provide any stool and/or did not provide sufficient data. Finally, another 27 patients (84.4%) were included for further analyses that observed alpha diversity, beta diversity, and composition of gut microbiota during the study period.

### 3.1. Demographic and Clinical Characteristics

The clinical characteristics of the included patients are shown in Table 1. The middle-aged group included 18 patients (7 females) and the late-life group 14 patients (10 females). There were no significant differences between the two groups in sex, Body Mass Index (BMI), HAM-D, HAM-A, GI symptoms of HAM-A, Chao1 index, or Shannon index at baseline.

### 3.2. Microbial Features and Their Changes during Treatment

**Figure 2:**
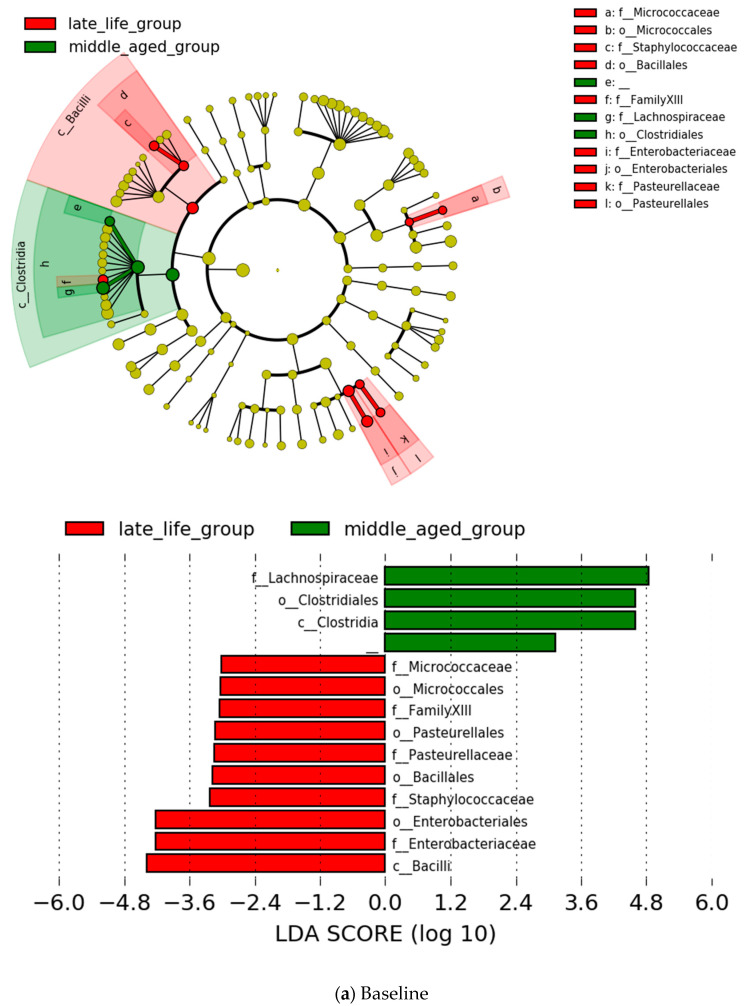

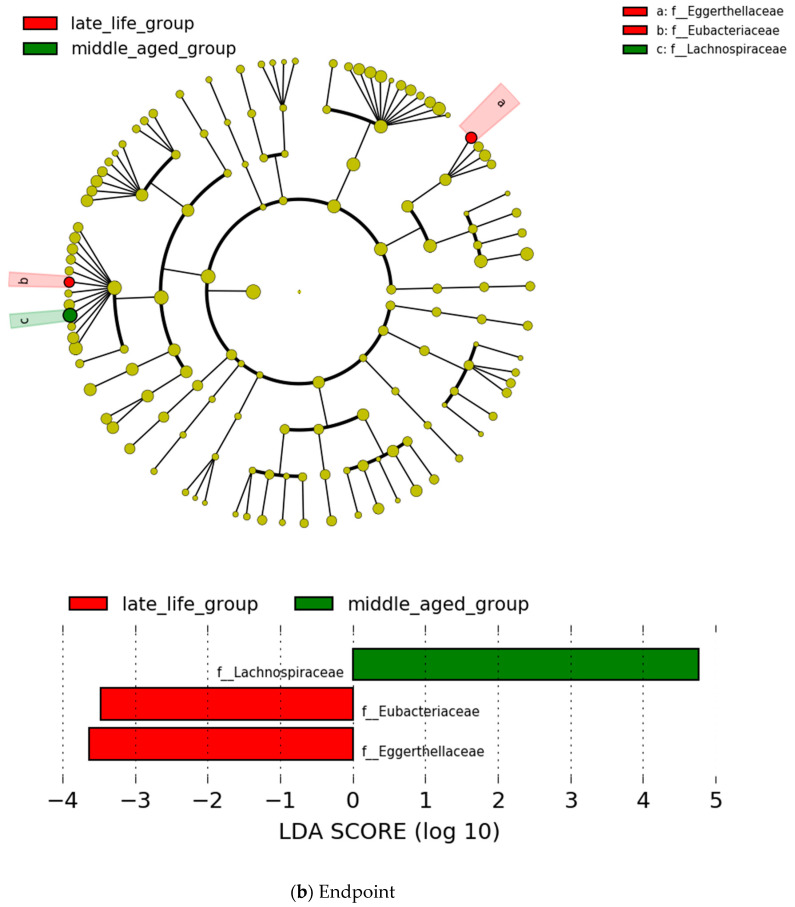
Specific bacterial contributor to the late-life (red) and middle-aged (green) groups by the LEfSe (Linear discriminant analysis (LDA) effect size) analysis at family level. Only LDA thresholds of >3.0 as determined by LEfSe are shown. Notes: “f” = family level, “c” = class level, and “o” = order level.

At the family level, five types of microbiota showed a significantly higher prevalence at baseline in the late-life group in comparison with the middle-aged group; the increase in two bacterial taxa was found to be representative at the endpoint in this group (Figure 2). In the middle-aged group, Lachnospiraceae showed a higher relative abundance at both baseline and endpoint compared with the late-life group (Figure 2). These differences did not survive after the FDR correction (*p* > 0.1).

**Figure 3:**
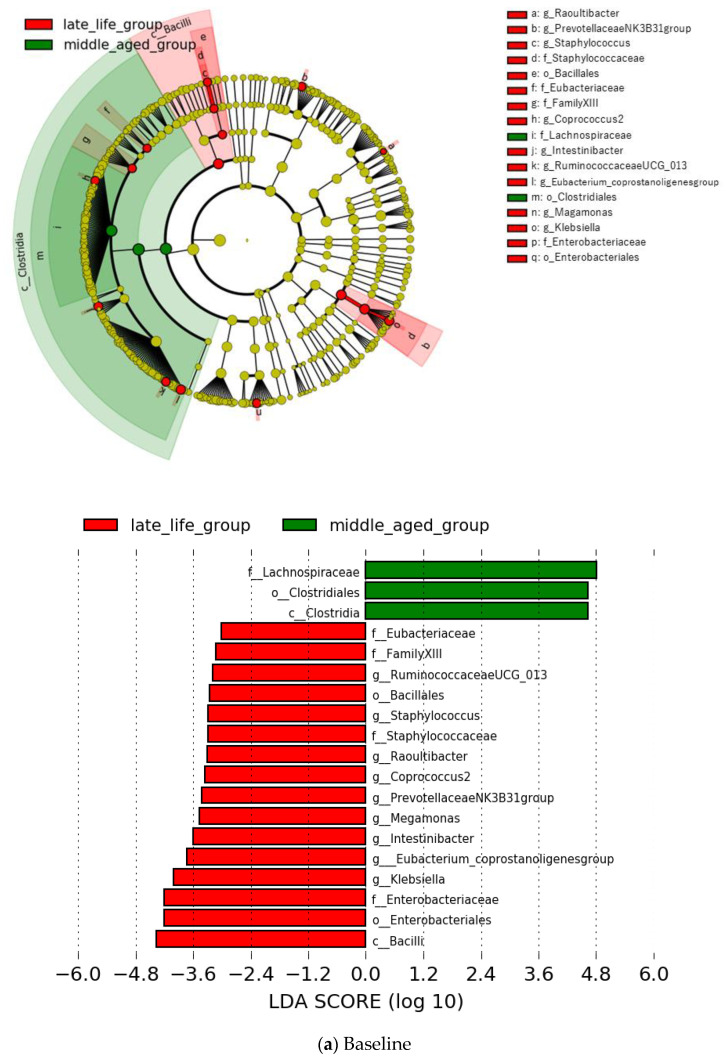

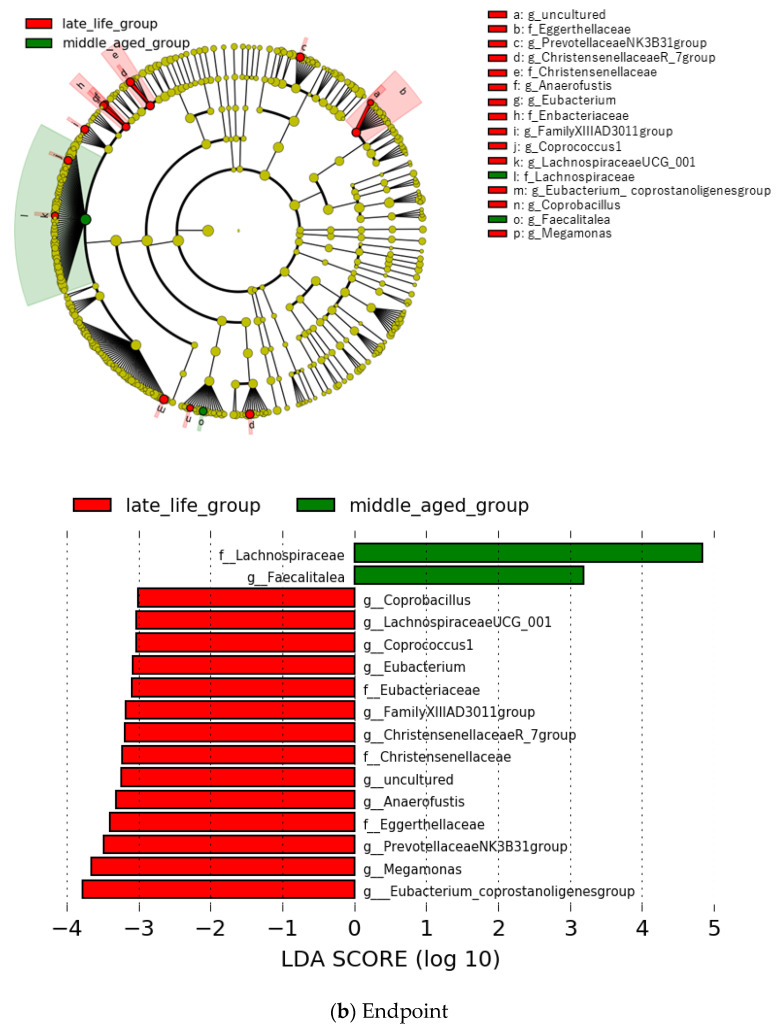
Specific bacterial contributor to the late-life (red) and middle-aged (green) groups by the LEfSe analysis (LDA >3.0) at genus level. Notes: “f” = family level, “g” = genus level, “c” = class level, and “o” = order level.

At the genus level, nine types of microbiota showed a significantly higher prevalence at baseline in the late-life group than in the middle-aged group. Among the increased levels of these bacterial taxa, *Megamonas*, *Prevotellaceae NK3B31 group* were found to be representative at the endpoint in the same group (Figure 3). None of the microbes showed significantly higher relative prevalence at baseline in the middle-aged group than in the late-life group, although *Faecalitalea* displayed a higher relative prevalence at the endpoint in this group (Figure 3). However, all the significances at the genus level did not survive after the FDR correction (*p* > 0.1).

We found no significant changes in the microbiota relative abundance after usual treatment (BL and T1 and BL and T2) at the family and genus levels within both the late-life and the middle-aged groups.

### 3.3. Baseline and Changes of Alpha Diversity

**Figure 4:**
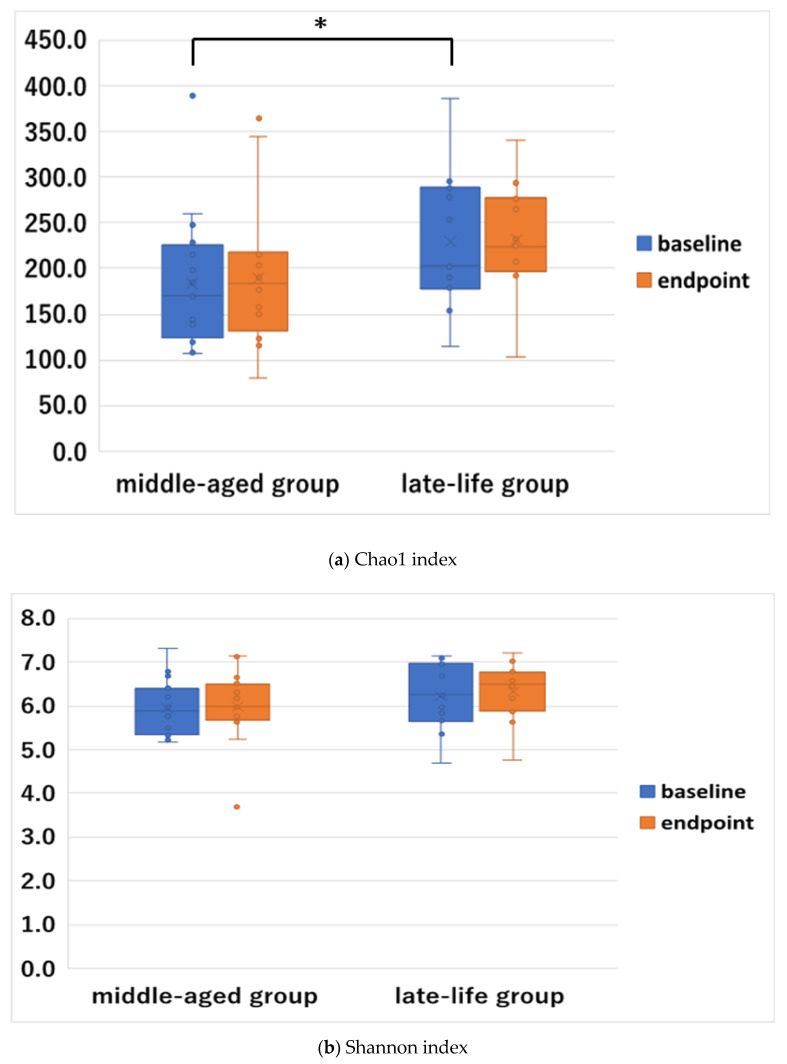
Microbiota alpha diversity between baseline (blue) and endpoint (orange) after the usual treatment in the late-life (*n* = 11) and middle-aged (*n* = 16) groups; * *p* < 0.05. Notes: The box signifies the upper (Q3) and lower (Q1) quartiles. The median is represented by a line and the mean by an X within each box. The whiskers extend up from the Q3 quartiles to the maximum data that are less than or equal to 1.5 times the interquartile range (IQR) and down from the Q1 quartile to the minimum data that are larger than 1.5 times the IQR. Values outside this range are considered to be outliers and are represented by dots.

At baseline, the late-life group showed a higher diversity in the Chao1 index compared with the middle-aged group (Figure 4), while there were no significant differences in the Shannon index (Figure 4). This result remained significant after the FDR correction (*p* = 0.044). After the usual treatment, these indices did not statistically differ from baseline to endpoint within each group (Figure 4).

Between the GI-present and GI-absent groups, the Chao1 and Shannon indices showed no significant differences at baseline (Supplementary Figure S1). Following the usual treatment, these indices did not statistically differ from baseline to endpoint within each group (Supplementary Figure S1).

### 3.4. Microbial Features and GI Symptoms in the Late-Life Group

**Figure 5:**
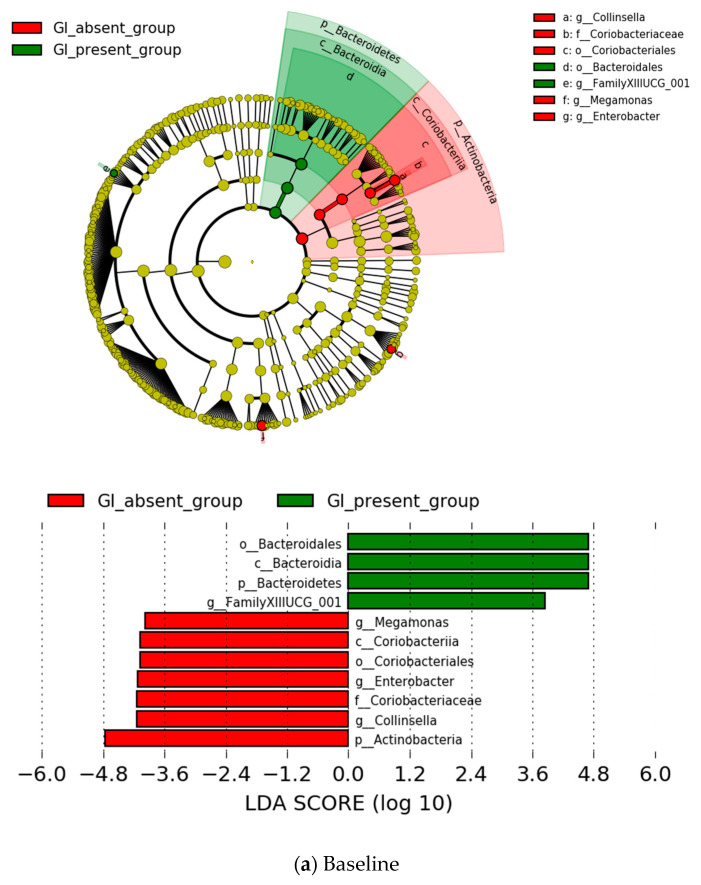

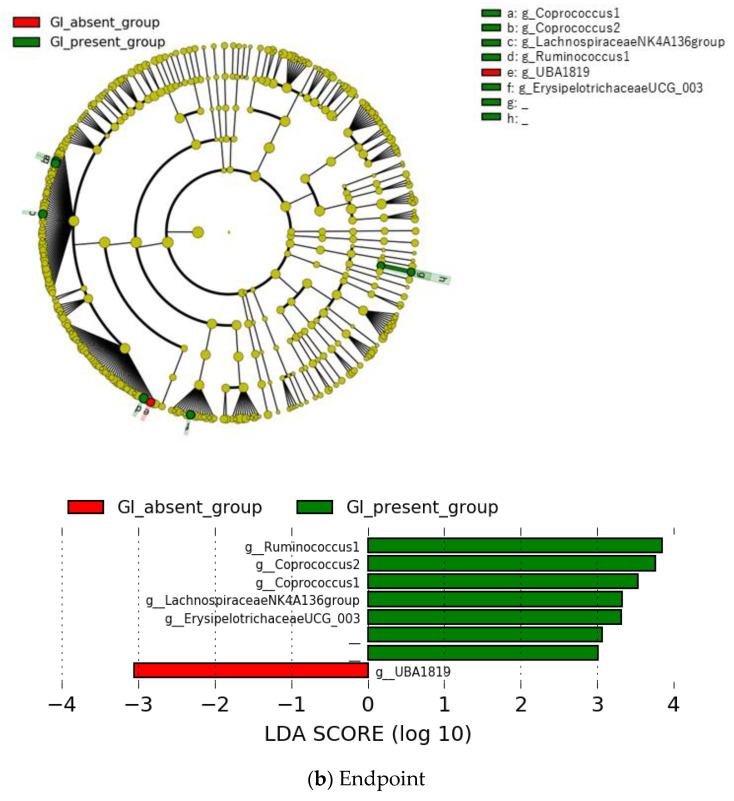
Specific bacterial contributor to GI-present symptom (*n* = 8, green) and GI-absent symptom (*n* = 6, red) groups by the LEfSe analysis (LDA >3.0) at genus level in the late-life group. Notes: “f” = family level, “g” = genus level, “c” = class level, and “o” = order level.

In the late-life group, we divided it into the two subgroups using the GI symptoms of the HAM-A. At the genus level, *Family XIII UCG-001* showed a significantly higher prevalence at baseline in the GI-present group in comparison with the GI-absent group; the increase of five bacterial taxa was found to be representative at endpoint in the former group (Figure 5). Among the increased levels of these bacterial taxa, none of the microbes were observed to be representative both at baseline and endpoint in this group (Figure 5). These differences of the *Family XIII UCG-001*, *Ruminococcus 1*, *Coprococcus 1*, and *Lachnospiraceae NK4A136 group* remained significant after the FDR correction. However, they did not correlate with GI symptoms.

### 3.5. Baseline Beta Diversity

As shown in Supplementary Figure S2, ANOSIM with permutations confirmed no significant separation of groups in the weighted and unweighted UniFrac distances, indicating that there were no clear differences in the structure of the bacterial community between the late-life group and the middle-aged group.

### 3.6. Relationship between the Severity of Depression and Microbial Features

In the late-life-group, family Eubacteriaceae and genus Eubacterium showed negative correlations with the scores of HAM-D (r = −0.371, *p* = 0.004; r = −0.265, *p* = 0.045, respectively), while they did not survive after the FDR correction. In the middle-aged-group, genus Faecalitalea showed a positive correlation with the scores of HAM-D (r = 0.265, *p* = 0.045). However, the significance was lost after the FDR correction.

## 4. Discussion

Age-dependent alterations in the composition of gut microbiota have previously been reported both in preclinical studies [23,24] and clinical studies [4,6,25,26]. The present study is the first to examine the relationships between age and compositional changes of gut microbiota as well as their relationship and GI symptoms in patients with MDD. Our main findings were as follows: (1) there were differences in the abundance of several bacterial taxa at baseline and/or endpoint between the late-life and middle-aged groups; while these differences did not survive after the FDR correction, there were no significant changes in gut microbiota composition over the course of treatment within the groups; (2) the late-life group showed significantly higher diversity in the Chao1 index at baseline compared with the middle-aged group after the FDR correction, and (3) there were significant differences in the abundance of several bacterial taxa at baseline and endpoint in the late-life group between the GI-present and GI-absent groups. However, there were no significant correlations between these bacterial taxa and scores of GI symptoms.

Here, depending on the age of patients with MDD, the following taxa in the late-life group were prominent compared to those in the middle-aged group; that is, 14 representative taxa (five families and nine genera) at baseline and 13 taxa (two families and 11 genera) at endpoint were increased in the late-life group, while family Lachnospiraceae both at baseline and endpoint and genus *Faecalitalea* at endpoint were increased in the middle-aged group. Notably, the abundance of the genera *Coprococcus*, *Prevotellaceae NK3B31 group*, *Megamonas*, and *Eubacterium coprostanoligenes group* was increased both at baseline and endpoint in the late-life group. *Coprococcus* is a genus of bacteria and produces butyric acid, which is one of the short-chain fatty acids (SCFAs) [27]. As stated previously, SCFAs could play a protective role against depression [28,29]. However, some previous studies demonstrated that the abundance of the genus *Coprococcus* was decreased in middle-aged patients with MDD, not in the late-life patients, compared to healthy controls (HCs) [30,31,32,33]. In addition, regarding genus *Prevotella*, it mainly produces propionic acid, which is one of the SCFAs [34] and it is known to be associated with glucose metabolism [35,36,37,38]. A previous review [39] noted inconsistent findings on *Prevotella* in patients with MDD. In the two previous studies by Lin et al. [40] and Liu et al. [31], a higher proportion of *Prevotella* was found in middle-aged patients with MDD compared to HCs, which is consistent with our finding. On the other hand, a lower abundance of *Prevotella* was reported in young and middle-aged patients with MDD compared to HCs in the study by Jiang et al. [41] and Kelly et al. [42], respectively. Similarly, regarding genus *Megamonas*, it produces propionic acid, which is one of the SCFAs [43]. A previous review [39] also noted inconsistent findings on *Megamonas* in patients with MDD. Consistent with our finding, Jiang et al. [41] reported a higher relative abundance of *Megamonas* in young patients with MDD, not in the late-life patients, compared to HCs; however, two studies showed lower relative abundances of *Megamonas* in middle-aged patients with MDD, not in the late-life patients, compared to HCs [31,33]. These discrepancies might be a result of differences in genetic analysis methods (e.g., 16S rRNA sequencing, region, pipeline analysis, or database) and age of target patients (i.e., young, middle-aged, and late-life patients). Taken together with the previous studies and our results, it remains unclear whether several gut microbiota that showed significant differences in the late-life group may be specific to late-life MDD or aging. Further studies examining the impact of age on the composition of gut microbiota will advance an understanding of the pathology of MDD.

Interestingly, we found a significant difference in the alpha diversity in the Chao1 index between the late-life and middle-aged groups at baseline; the late-life group showed significantly higher diversity compared to the middle-aged group. In line with our finding, previous studies reported that the alpha diversity of the gut microbiota in human adults increases with age [4,44]. One review study by de la Cuesta-Zuluaga et al. [44] based on four cohorts in healthy adults between 20 and 69 years of age reported a positive trend between age and alpha diversity in the sequence variant (SV) richness and the Shannon index. Further, a cross-sectional study by Odamaki et al. [4] reported that a positive correlation was established between age and alpha diversity in the Chao1 index, number of observed species, PD whole tree index, and the Shannon index in healthy Japanese subjects from the elderly to the centenarian stage. On the other hand, we did not find any significant differences in the alpha diversity in the Chao1 or Shannon indices between the GI-present and GI-absent groups. One possible explanation for this finding could be the differences in the type of alpha diversity (e.g., components of richness, evenness, or phylogenetic diversity). Although very few studies investigated the association between alpha diversity and GI symptoms in patients with psychiatric diseases including MDD, these relationships were investigated in patients with inflammatory bowel disease (IBD) in previous studies [45,46]. However, findings of these studies in this population were inconsistent. That is, Tong et al. [46] reported that phylogenetic diversity, not the Chao1 or Shannon indices, was lower in patients with an active state of IBD as well as a quiescent state of IBD; by contrast, Shutkever et al. [45] demonstrated that phylogenetic diversity was higher in patients with a quiescent state of IBD compared to patients with an active state of IBD. Thus, future studies are warranted focusing on the relationship between microbial diversity and GI symptoms in patients with MDD.

We also determined that several bacterial taxa were associated with GI symptoms at baseline and endpoint in the late-life group; a higher proportion of *Family XIII UCG-001* at baseline and higher abundances of *Ruminococcus 1*, *Coprococcus 1*, *Lachnospiraceae NK4A136 group* at endpoint were observed in the GI-present group in comparison with the GI-absent group. Although there is little information on *Family XIII UCG-001* and *Lachnospiraceae NK4A136 group* in previous studies, it is reported that they were associated with depression-like behavior in mice [47]. Further, the proportion of *Coprococcus* was decreased in middle-aged patients with MDD, not in the late-life patients, compared to HCs as stated above [30,31,32,33]. Likewise, a previous review [39] noted that a decrease in the prevalence of *Ruminococcus* was found in middle-aged and young patients with MDD [31,41], not in the late-life patients, compared to HCs. On the other hand, these four bacterial taxa related with GI symptoms are a member of phylum Firmicutes, and it is considered to be one of the two major phyla present in healthy subjects [48]. In agreement with our finding, an increase in the composition of Firmicutes was found in patients with irritable bowel syndrome (IBS) [49] and inflammatory bowel disease (IBD) [50], while the finding was inconsistent in patients with MDD in a previous review [39]. Given these previous studies and our findings, including the fact that we did not find any bacterial taxa associated with GI symptoms in the middle-aged group, future research examining the impact of age on the composition of specific gut microbiota and their relationship with GI symptoms will aid in understanding their effectiveness in different age groups of MDD patients.

This study has several limitations. First, we did not consider the impact of daily diet on the composition of gut microbiota in patients with MDD. It is generally recognized that diet could affect the composition of gut microbiota and their function [51]. In the current study, daily meals may have affected our findings, especially in the outpatients, which should be addressed by future research. Second, we did not evaluate the effects of prescription medications taken before participating in the present study, while we reported the effects of psychotropics on the gut microbiota in patients with MDD elsewhere from the original study [11]. In particular, antidepressants are known to exhibit antimicrobial effects [52,53]. Third, our findings of this study may have problems with reproducibility due to the small sample size. In this study, there were trend-toward differences in HAM-D and HAM-A scores between the late-life and the middle-aged groups. Thus, large cohort studies are needed in the future to avoid beta errors. Fourth, there is a lack of age-matched healthy controls or age-matched controls with GI symptoms in this study. Fifth, we did not take into account the time difference in the stool sample collection between inpatients and outpatients, as we conducted this study in an ordinary clinical setting, which may have affected the results. For inpatients, the timing of sample collection was clearly determined; however, for outpatients, the timing of sample collection depended on the visit interval. Finally, among patients with GI symptoms, selection bias may have occurred, considering the possibility that patients of high interest in this study may have participated.

## 5. Conclusions

Despite the aforementioned limitations, the current study found that specific bacterial taxa had higher relative abundances in the late-life group than in the middle-aged group, while these differences did not survive after the FDR correction; moreover, a diversity in the Chao1 index at baseline was significantly higher in the late-life group than in the middle-aged group after the FDR correction. We further found possible microbial taxa related to GI symptoms in patients with late-life depression. Our study is a preliminary study for future extensive studies to examine the relationship between brain–gut interaction and age in MDD. Thus, the present study warrants further research to clarify potential impacts of age and GI symptoms on specific bacterial taxa in patients with MDD to develop an age-stratified treatment of MDD.

## Figures and Tables

**Table 1:** Clinical characteristics of the included patients.

Characteristics	Middle-Aged Group (*n* = 18)	Late-Life Group (*n* = 14)		
	n	n	χ^2^	*p*
Gender (Female/Male)	7/11	10/4	0.67	0.087
	**Mean (SD)**	**Mean (SD)**	***t***	***p***
Age (Years)	41.1 (10.1)	73.5 (8.7)	9.242	**<0.001**
BMI (Kg/m^2^)	23.1 (5.0)	21.2 (3.1)	1.256	0.219
HAM-D	17.8 (7.0)	12.2 (8.9)	1.916	0.064
HAM-A	17.2 (7.9)	11.7 (8.0)	1.860	0.072
HAM-A GI symptoms	1.3 (1.0)	1.2 (0.9)	0.132	0.896
Chao 1 Index	186.7 (70.7)	246.4 (76.6)	2.416	**0.022**
Shannon Index	6.0 (0.6)	6.4 (0.8)	1.441	0.160

SD, standard deviation; BMI, Body Mass Index; HAM-D, Hamilton Rating Scale for Depression; HAM-A, Hamilton Rating. Scale for Anxiety; GI, gastrointestinal.

## Data Availability

The data presented in this study are available on reasonable request from the corresponding author. The data are not publicly available.

## Supplementary Materials

The following supporting information can be downloaded at: https://www.mdpi.com/article/10.3390/jpm12111827/s1, Figure S1: Microbiota alpha diversity between baseline (blue) and endpoint (orange) after the usual treatment in the patients with GI symptoms (GI-present group) and without GI symptoms (GI-absent group). Figure S2: Microbiota beta diversity of the samples in the middle-aged group (blue) and the late-life group (orange) by PCoA (Principal coordinate analysis) based on unweighted (a) and weighted (b) UniFrac distances and analysis of similarity (ANOSIM) tests.
