# Supplementary material for: The Potential Impact of Age on Gut Microbiota in Patients with Major Depressive Disorder: A Secondary Analysis of the Prospective Observational Study
Source: J Pers Med. 2022 Nov 3;12(11):1827. doi: 10.3390/jpm12111827 (PMC9697470; doi:10.3390/jpm12111827)
Supplement: Supplementary file 1 [file jpm-12-01827-s001.zip › jpm-1809103-supplementary.pdf]

Supplementary Materials:

The potential impact of age on gut microbiota in patients with major depressive disorders: A prospective observational study

Katsuma Miyaho, Kenji Sanada, Shunya Kurokawa, Arisa Tanaka, Tomoyuki Tachibana, Chiharu Ishii, Yoshihiro Noda, Shinichiro Nakajima, Shinji Fukuda, Masaru Mimura, Taishiro Kishimoto, Akira Iwanami

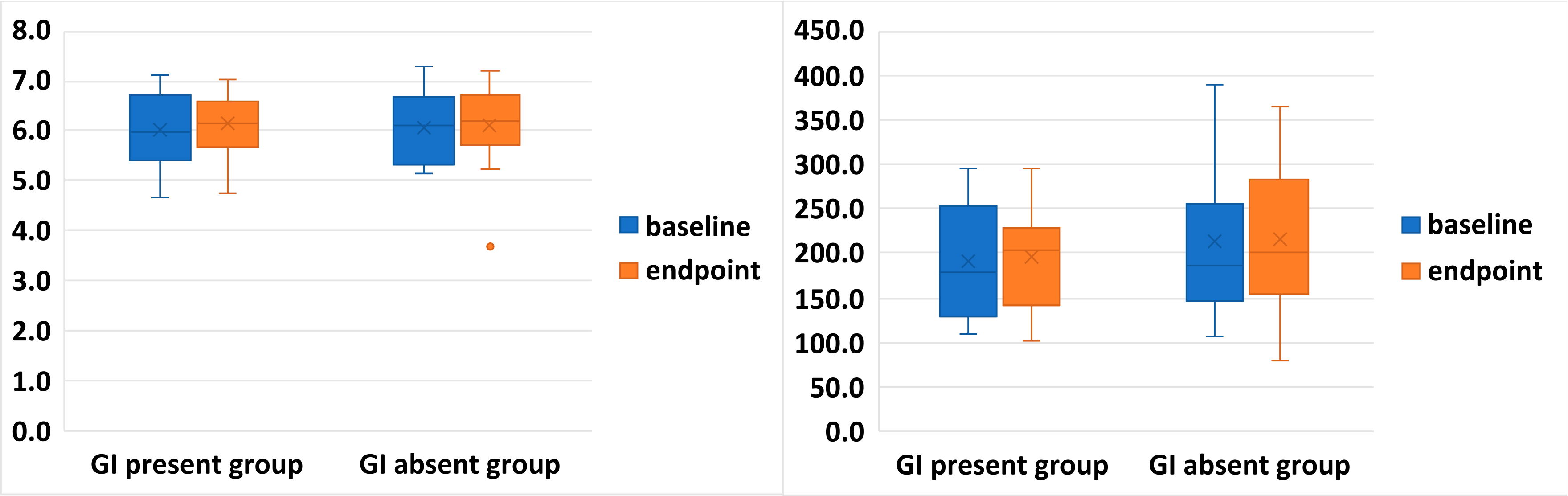

(a) Shannon index **(b) Chao1 index**

Figure S1. Microbiota alpha diversity between baseline (blue) and endpoint (orange) after the usual treatment in the patients with GI symptoms (GI present group) and without GI symptoms (GI absent group). **Notes:** The box signifies the upper (Q3) and lower (Q1) quartiles. The median is represented by a line and the mean by a X within each box. The whiskers extend up from the Q3 quartiles to the maximum data that is less than or equal to 1.5 times the interquartile range (IQR) and down from the Q1 quartile to the minimum data that is larger than 1.5 times the IQR. Values outside this range are considered to be outliers and are represented by dots.

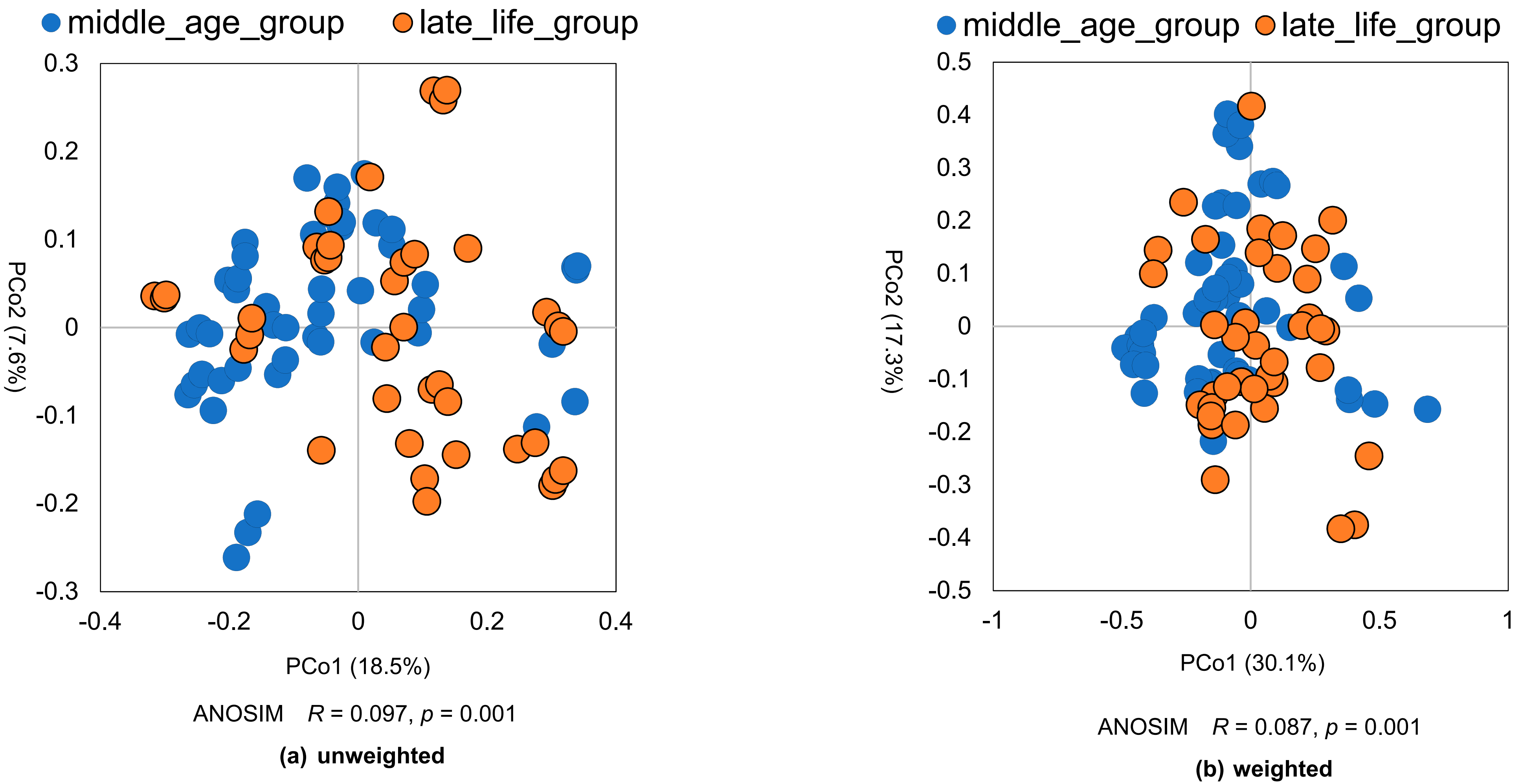

Figure S2. Microbiota beta diversity of the samples in the middle-aged group (blue) and the late-life group (orange) by PCoA (Principal coordinate analysis) based on unweighted (**a**) and weighted (**b**) UniFrac distances and analysis of similarity (ANOSIM) tests.
